# Supplementary figures and images for: Tissue of origin characterization of cell free DNA in seminal plasma: Implications for new liquid biopsies
Source: PLoS One. 2025 Mar 31;20(3):e0317712. doi: 10.1371/journal.pone.0317712 (PMC11957367; doi:10.1371/journal.pone.0317712)

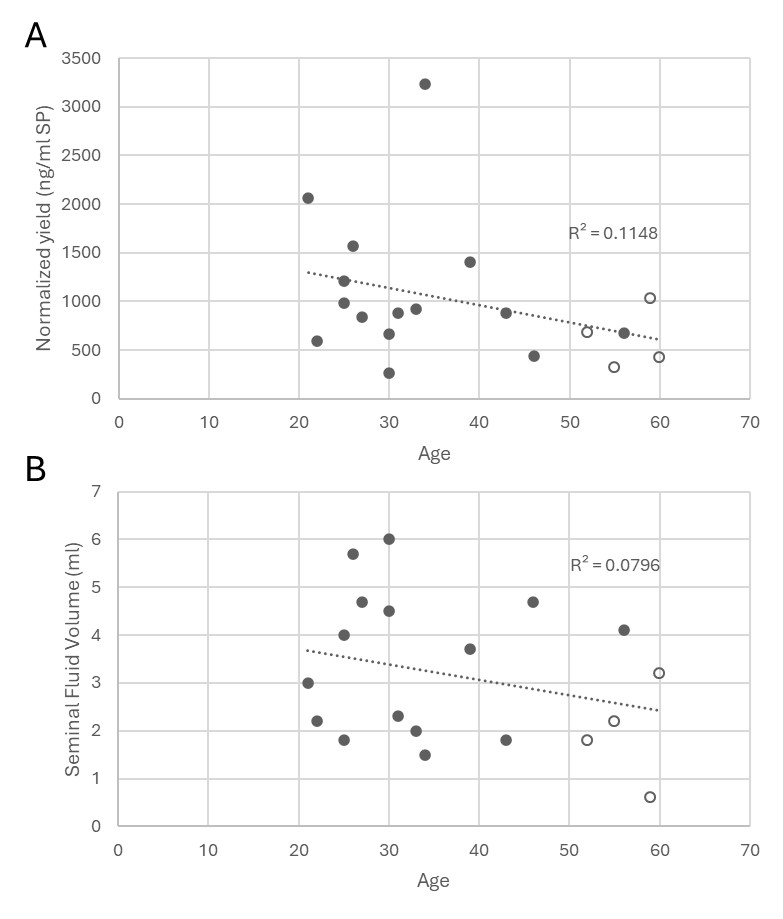

Supplement: S1 Fig — Solid circles are non-vasectomy subjects, open circles are vasectomy subjects. A: Normalized yield (ng/ml seminal plasma) versus age. B: Seminal fluid volume versus age. (JPG) [file pone.0317712.s001.jpg]

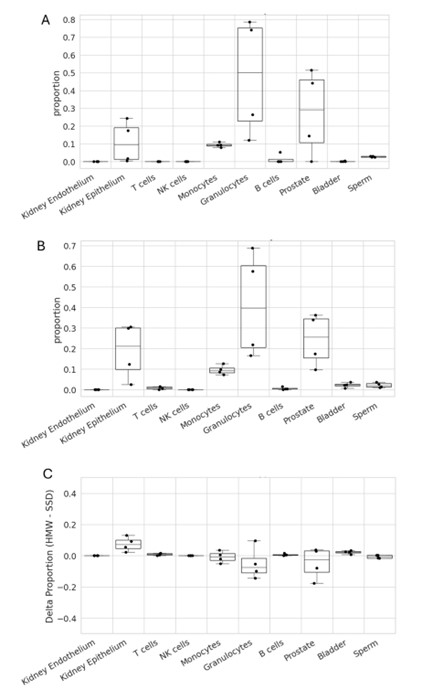

Supplement: S2 Fig — A: Tissue deconvolution results for size selected small cfDNA (SSD). B: Tissue deconvolution results for high molecular weight cfDNA (HMW). C: Difference in proportion of signal between HMW and SDD (n = 4). It is important to note that the algorithm used in the tissue deconvolution sums the signal to add to 1 (i.e.,100%), therefore while it appears that the vasectomy subjects have a lot more signal from the various somatic cells, this is driven by the absence of any sperm signal. (JPG) [file pone.0317712.s002.jpg]

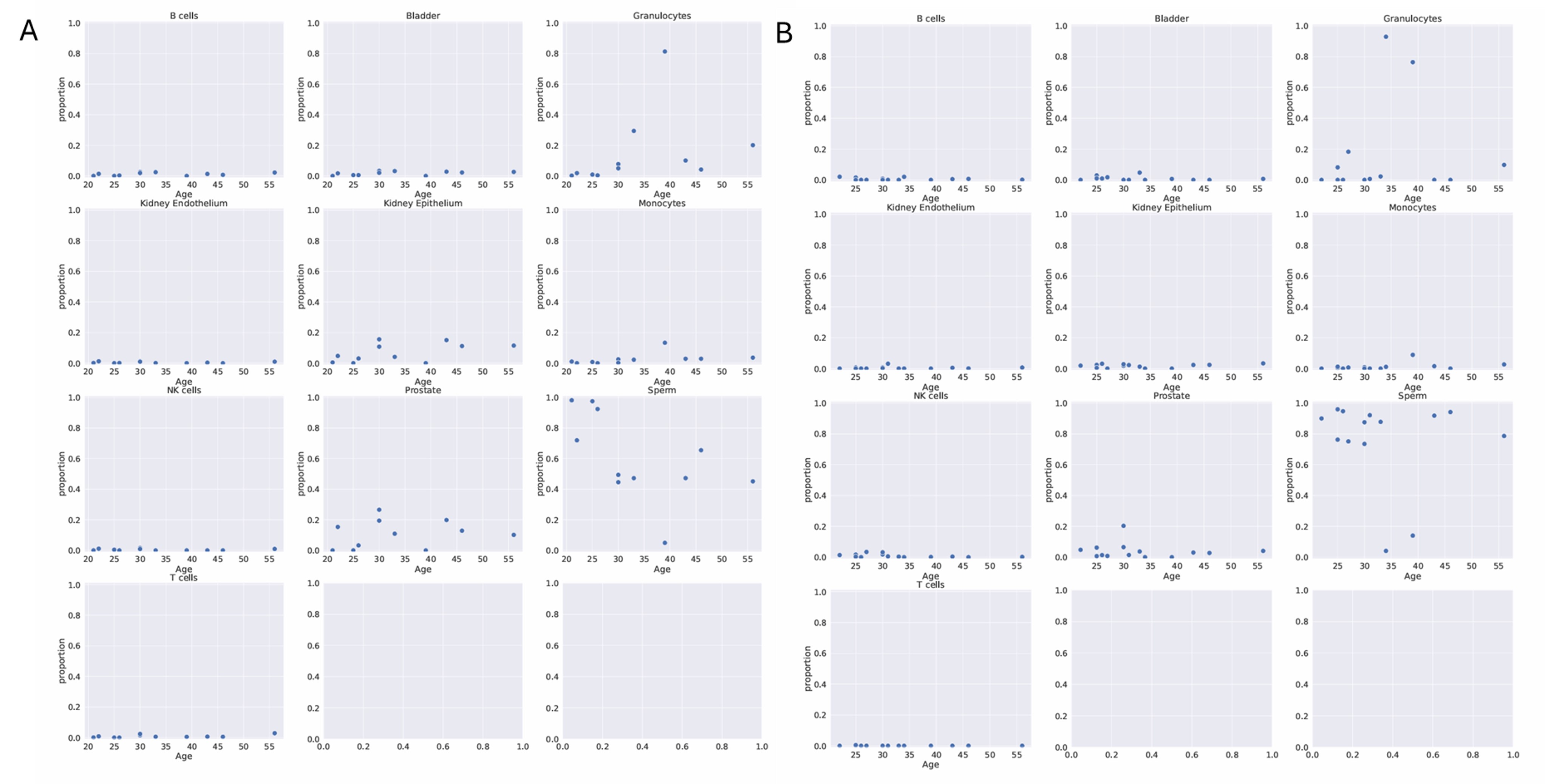

Supplement: S3 Fig — A: HMW tissue deconvolution by age. B: SSD tissue deconvolution by age. (JPG) [file pone.0317712.s003.jpg]

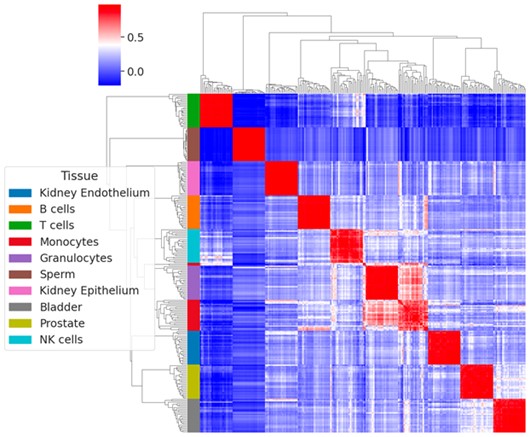

Supplement: S4 Fig — Pearson correlation followed by clustering of the n = 25 methylation markers for each reference tissue show strong agreement within their tissue and mostly poor correlation across tissues. An exception is the slight intermixing of the granulocyte/monocyte marker clusters, which can be explained by their similar developmental origins. (JPG) [file pone.0317712.s004.jpg]

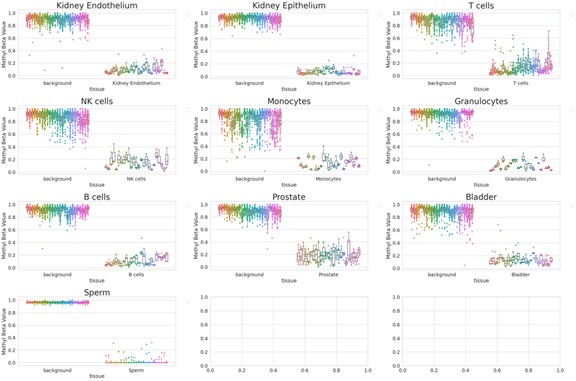

Supplement: S5 Fig — Each graph represents methylation values for a tissue/cell type specific set of hypomethylation markers. Shown are methylation values for background (all other tissue/cell types) and the tissue/cell type of interest for all samples in the reference dataset. (JPG) [file pone.0317712.s005.jpg]

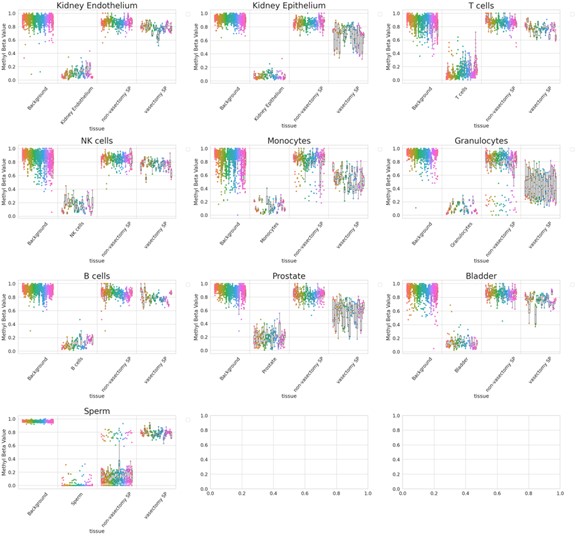

Supplement: S6 Fig — Each graph represents methylation values for a tissue/cell type specific set of hypomethylation markers. Shown are methylation values for background (reference dataset), tissue of interest (reference dataset), non-vasectomy seminal plasma (SP) samples and vasectomy SP samples. Note that the methylation profile for most of the non-vasectomy SP more closely matches sperm more so than any other tissue type, illustrating sperm to be the most prominent cell type. In vasectomy samples, prostate, granulocytes and monocytes markers appear hypomethylated compared to background tissues, suggesting the presence of DNA from these cell types within these samples. (JPG) [file pone.0317712.s006.jpg]

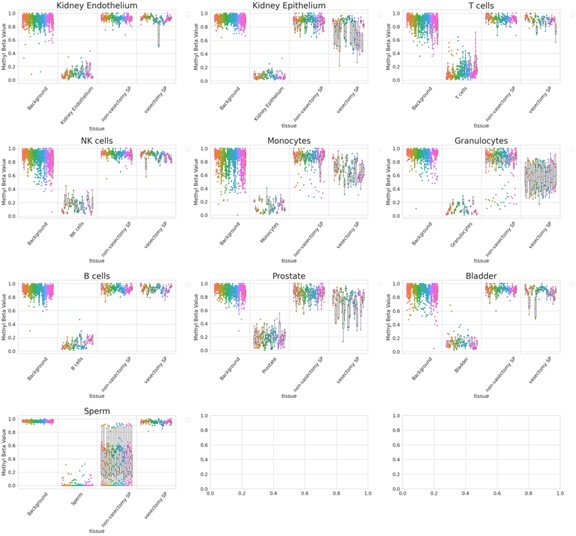

Supplement: S7 Fig — Each graph represents methylation values for a tissue/cell type specific set of hypomethylation markers. Shown are methylation values for background (reference dataset), tissue of interest (reference dataset), non-vasectomy seminal plasma (SP) samples and vasectomy SP samples. Note the very large variability in signal for sperm markers in non-vasectomy samples demonstrating a large range of sperm signal present. (JPG) [file pone.0317712.s007.jpg]

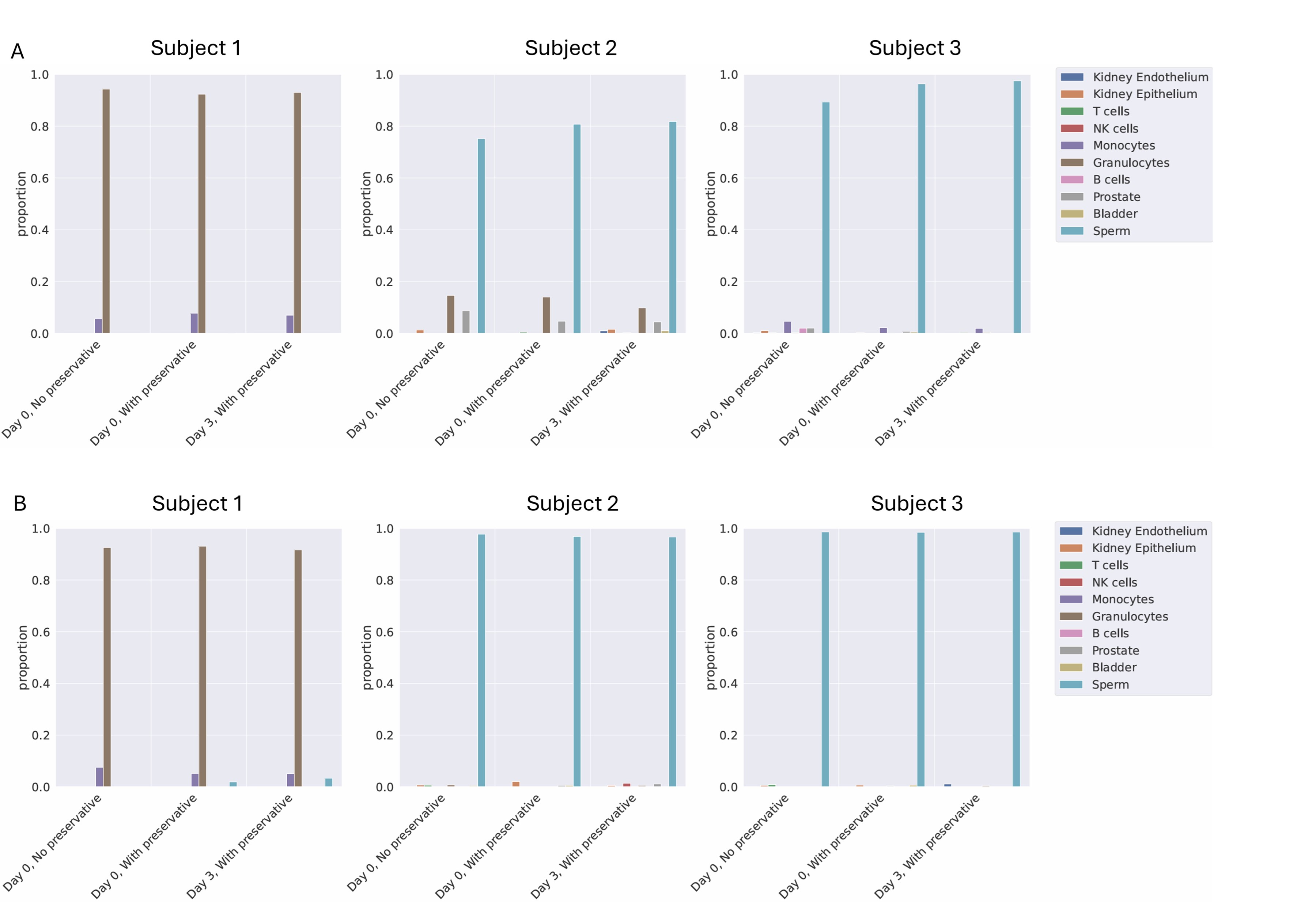

Supplement: S8 Fig — Graphs show tissue deconvolution results for 3 subjects at day 0 with and without preservative and at day 3 in the oven with preservative. A: HMW results: Subject 1 has predominantly granulocyte signal with some monocyte signal, while subjects 2 and 3 have predominantly sperm signal. On day 0 for all the 3 subjects the results look very similar whether preservative was added or not. Additionally, for all 3 subjects, the relative proportion of each cell type is similar on day 3 with preservative to day 0. B: SSD results: Subject 1 again has predominantly granulocyte signal with some monocyte signal, while subjects 2 and 3 have almost 100% sperm signal at day 0 with and without preservative and at day 3 with preservative. (JPG) [file pone.0317712.s008.jpg]
