# Supplementary material for: Tissue of origin characterization of cell free DNA in seminal plasma: Implications for new liquid biopsies
Source: PLoS One. 2025 Mar 31;20(3):e0317712. doi: 10.1371/journal.pone.0317712 (PMC11957367; doi:10.1371/journal.pone.0317712)
Supplement: Attachment — Submitted filename: Response_to_Reviewers_auresp_2.docx [file pone.0317712.s016.docx]

Dear Dr. Alexis G. Murillo Carrasco,

Thank you for your response regarding our manuscript. We have gone through the style requirements guide and have updated the manuscript accordingly, including updating the Refences with the correct style. We have also removed the funding source from the acknowledgements section. The funder, Fellow Health Inc., is the employer of all authors, and funded salaries, sample collection, reagents, instrumentation and sequencing costs.

We also appreciate the comments received from the 3 reviews. Below is a description of the changes made to address each point raised by them.

**Reviewer #1:** First, there were samples that were preserved and other were not. This might affect the results, and it is not something the authors seem to be considering in their subsequent analysis.

**Response:** We have now included Figure S8 which shows the tissue deconvolution results for 3 subjects with and without preservative on day 0. Hopefully this addresses the reviewer’s concern and illustrates that tissue deconvolution results for the same subject are similar on day 0 with and without preservative. Figure S8 also shows that with preservative on day 3 in the oven, the tissue deconvolution results still look similar to day 0. One would not expect the results to look the same on day 3 without preservative due to cell lysis; the Tapestation images clearly show that there is a much higher yield of DNA after 3 days in the oven when there is no preservative.

**Reviewer #1:** Other factors that could be affecting the results are i) the age of the patients included, which ranges from 20-60 years old; and ii) the n of each group.

**Response:** We have added additional graphs to the supplementary information regarding patient age. Figure S1 shows normalized cfDNA yield and total semen volume by age. Figure S3 shows tissue deconvolution results by age. The number within each age category can clearly be seen in both S1 and S3.

**Reviewer #1:** In addition, the preservative composition used is not detailed at all and, therefore, the reproducibility of the results is compromised.

**Response:** The preservative is proprietary to Fellow Health Inc. who funded this work, so we are unable to provide the composition. However, we would be willing to provide others with the preservative for reproduction of the results. Additionally, since we have illustrated that the tissue deconvolution results are the same with and without preservative on day 0, others could simply look at tissue deconvolution without preservative on day 0.

**Reviewer #2:** The authors mention "The sequencing data will be available after acceptance on NIH BioProject.
<https://www.ncbi.nlm.nih.gov/bioproject/PRJNA1162531>" However, for code reproducibility it would be important for the authors to include their code on Github or a similar page, as well as a full analytical pipeline so their results can be reproduced.

**Response:** Scripts needed to run the methylation tissue deconvolution have been uploaded to github at <https://github.com/meetfellow/fellow-genomics>. This link has also been added to the manuscript under the methylation tissue deconvolution methods section.

**Reviewer #2:** It is important to validate identified prostate/kidney/granulocytes markers with other datasets. DNAm is a key marker of tissue identity that tends to overlap with DNase I hotspots (another key marker of tissue identity) for the tissue of origin. To confirm that the identified sites are genuinely from the tissues listed (prostate, etc.) and have biological roles in these tissues it would be important to show preferential overlap with DNase I hotspots for prostate/kidney/granulocytes. This could be done using eFORGE (<https://eforge.altiusinstitute.org/>, there are probably a range of sites that overlap between the DNAm markers identified here and the EPIC array), or the eFORGE table S3 and S4 panel of blood and kidney tissue-specific differentially methylated positions (tDMPs). Other analyses using other chromatin datasets such as histone marks or chromatin states could also help clarify this issue.

**Response:** We validated the potential biological role of these tissue specific methylation markers using ENCODE DNase I hypersensitivity data. Using n=102 ENCODE datasets from 7 different matched tissues, we looked for proximity between the methylation markers and DNase I hypersensitive sites. We found that, on average, 45.0% of methylation hypo markers had a DNase I site from the marker’s tissue within 250bp, compared to an average of 3.7% for non-tissue DNase I sites. These results are now illustrated in Table S3 and discussed in the results section.

**Reviewer #2:** Table S2 seems to be missing statistics/p-value and other information used in ranking top DNA methylation (DNAm) positions.

**Response:** We have added Table S2 to address this comment. For each marker it shows the tissue it marks, as well as its separation score (described in methods) that we used to rank markers and select the top 25.

**Reviewer #3:** Abstract Pg 1 not clear
While sperm was the predominant signal in most men without vasectomies, the proportion of predicted prostate contribution reached as high as 26.5% in the HMW fraction. In other subjects without vasectomies, granulocyte cfDNA made up most of the signal.

**Response:** We have modified as follows: “While sperm was the predominant signal in most men without vasectomies, granulocyte cfDNA made up most of the signal in two of the non-vasectomy subjects. Unexpectedly, the proportion of prostate signal reached as high as 26.5% in the HMW fraction in non-vasectomy subjects”.

**Reviewer #3:** What is the preservative?

**Response:** The preservative is proprietary to Fellow Health Inc. who funded this work, so we are unable to provide the composition. However, we would be willing to provide others with the preservative for reproduction of the results. Additionally, since we have illustrated that the tissue deconvolution results are the same with and without preservative on day 0, others could simply look at tissue deconvolution without preservative on day 0.

**Reviewer #3:** What is the significance of using preservative immediately and leaving the sample for three days?

**Response:** If a test developer were to want to make an at home collection kit, they would need to ensure that the sample is stable during shipping. The significance of adding the preservative immediately and leaving it for 3 days was to demonstrate that the preservative can stabilize cfDNA (and prevent sperm lysis) if shipping back to the laboratory were to take 3 days. The following sentence was added to the manuscript, “Adding preservative immediately and waiting for 3 days before preparing seminal plasma best represents the scenario of an at home collection test.”

**Reviewer #3:** The english of the manuscript needs to be more lucid.

**Response:** We have modified the part of the abstract that was not clear to reviewer #3 to the following: “While sperm was the predominant signal in most men without vasectomies, granulocyte cfDNA made up most of the signal in two of the non-vasectomy subjects. Unexpectedly, the proportion of prostate signal reached as high as 26.5% in the HMW fraction in non-vasectomy subjects”. If there are other sentences or paragraphs that are not clear we can certainly re-write. The other two reviewers answered, “yes” to the question, “Is the manuscript presented in an intelligible fashion and written in standard English?” so we are not sure which parts of the manuscript are not “lucid”.

We also made some additional changes to Figures as summarized below:

1. Figure 3: For panels D and E we changed to color images as it was difficult to see the various different samples in grayscale.
2. Figure 4: For panel C, we reduced the range on the y-axis so that changes were more obvious (there was a lot of white space in the previous plot).
3. Figure S1, that is now S2: For panel C, we reduced the range on the y-axis so that changes were more obvious (there was a lot of white space in the previous plot).

Finally, we also corrected the subject and library numbers in the Materials and Methods section for methylation to match the correctly annotated Figure 4, its associated legend, and the results section. There were actually 15 SSD non-vasectomy libraries (not 14) and all 15 HMW samples had matching SSD samples.

We hope that with these changes, the manuscript is now suitable for acceptance.

Best,

Kim Clark-Langone
