# Supplementary material for: Tissue of origin characterization of cell free DNA in seminal plasma: Implications for new liquid biopsies
Source: PLoS One. 2025 Mar 31;20(3):e0317712. doi: 10.1371/journal.pone.0317712 (PMC11957367; doi:10.1371/journal.pone.0317712)
Supplement: Attachment — Submitted filename: Response to Reviewers_2.docx [file pone.0317712.s017.docx]

Dear Dr. Alexis G. Murillo Carrasco,

Thank you for your response regarding our manuscript. We also appreciate the additional comments received from reviewer #2. Please see below for our response to each comment.

**Reviewer #2:** Re "We found that, on average, 45.0% of methylation hypo markers had a DNase I site from the marker’s tissue within 250bp, compared to an average of 3.7% for non-tissue DNase I sites. These results are now illustrated in Table S3 and discussed in the results section." These results are encouraging but there are several sources of confounding that can be driving this including promoter content and CpG content/CpG Island density (hence the suggestion of using eFORGE). Can you adjust for these confounders or show that even a subset of CpGs are still enriched after adjusting for promoter content or CpG content/CpG Island density (with code on GitHub)?

**Response:** One of the major objectives of this study was to develop a tissue deconvolution approach that could be used with seminal plasma. Our methodology was based a Nature paper (Loyfer et al, 2023) whereby they determined potential tissue-specific biomarkers for use in liquid biopsies. We supplemented these datasets with data sourced from other studies to capture the full expected tissue content of seminal plasma (i.e. sperm). We realize that eFORGE can be useful in understanding tissue-specific relevance and potential functional roles of hypomethylated sites, but this was not one of the aims of this study. The studies that were the source of our reference methylation data have already explored much of the potential functional nature of such marks. We undertook a good faith analysis to satisfy the request of reviewer #2 and feel that further analysis is unwarranted as the relationship between DNase I sensitivity and DNA methylation is complex and context-dependent. We do not feel that the use of eFORGE would be suitable for our work, as this database has few overlapping tissues with our reference methylation data. It also uses methylation arrays, which have low overlap with our methylation enrichment panel, and as such we do not believe it appropriate for the suggested analysis. We also note that our methylation panel was not restricted to promoter regions but included a wide range of diverse genomic locations.

We have modified our updated language (in response to the original comment by reviewer #2) to remove the notion that we were “verifying” or “validating” tissue functionality as part of this work and are happy to completely remove any language relating to functionality given this was not an aim of this study. Again, the main objective was to establish a methodology for tissue deconvolution based on the Nature paper by Loyfer et al, 2023, not to demonstrate tissue functionality.

**Reviewer #2:** Please ensure that vectorial figures are included (vectorial and not bitmap) so they are readable in the PLOS ONE pdf. Current figures are grainy and including vectorial (or high resolution) images will greatly improve this work.

**Response:** We have modified images to meet PLOS One requirements.

We hope that with these changes, the manuscript is now suitable for acceptance.

Best,

Kim Clark-Langone
